# Phonotactic Constraints Are Activated across Languages in Bilinguals

**DOI:** 10.3389/fpsyg.2016.00702

**Published:** 2016-05-18

**Authors:** Max R. Freeman, Henrike K. Blumenfeld, Viorica Marian

**Affiliations:** ^1^Bilingualism and Psycholinguistics Research Group, Roxelyn and Richard Pepper Department of Communication Sciences and Disorders, Northwestern UniversityEvanston, IL, USA; ^2^Bilingualism and Cognition Laboratory, School of Speech, Language, and Hearing Sciences, San Diego State UniversitySan Diego, CA, USA

**Keywords:** bilingualism, phonology, epenthesis, parallel language activation, comprehension

## Abstract

During spoken language comprehension, auditory input activates a bilingual’s two languages in parallel based on phonological representations that are shared across languages. However, it is unclear whether bilinguals access phonotactic constraints from the non-target language during target language processing. For example, in Spanish, words with s+ consonant onsets cannot exist, and phonotactic constraints call for epenthesis (addition of a vowel, e.g., stable/*estable*). Native Spanish speakers may produce English words such as *estudy* (“study”) with epenthesis, suggesting that these bilinguals apply Spanish phonotactic constraints when speaking English. The present study is the first to examine whether bilinguals access Spanish phonotactic constraints during English *comprehension*. In an English cross-modal priming lexical decision task, Spanish–English bilinguals and English monolinguals heard English cognate and non-cognate primes containing s+ consonant onsets or controls without s+ onsets, followed by a lexical decision on visual targets with the /e/ phonotactic constraint or controls without /e/. Results revealed that bilinguals were faster to respond to /es/ non-word targets preceded by s+ cognate primes and /es/ and /e/ non-word targets preceded by s+ non-cognate primes, confirming that English primes containing s+ onsets activated Spanish phonotactic constraints. These findings are discussed within current accounts of parallel activation of two languages during bilingual spoken language comprehension, which may be expanded to include activation of phonotactic constraints from the irrelevant language.

## Introduction

Across many contexts and discourse situations, bilinguals activate both languages simultaneously, even when only one language is used overtly, a phenomenon known as parallel activation (e.g., Green, 1998; Dijkstra and van Heuven, 2002; Blumenfeld and Marian, 2007; Kroll et al., 2008; Shook and Marian, 2013). Bilinguals have previously demonstrated parallel activation of phonological (Marian and Spivey, 2003; Blumenfeld and Marian, 2007, 2013; Darcy et al., 2015), lexical (Linck et al., 2008; Bartolotti and Marian, 2012), semantic (Martín et al., 2010), and syntactic (Linck et al., 2008; Kootstra et al., 2012) information across their two languages. In the current study, we explore whether cross-linguistic activation of phonological structures extends to phonotactic constraints (i.e., legal ways for combining speech sounds) of the non-target language during spoken word comprehension in bilinguals. Specifically, we address the question: *Do Spanish–English bilinguals access Spanish phonotactic constraints during English comprehension?*

Phonotactic constraints can differ across languages, which may become a stumbling block for second language (L2) speakers during initial stages of L2 acquisition and use (e.g., Flege and Davidian, 1984). Specifically, language production studies suggest that when the phonology of the L2 does not align with or is not present in the native language (L1), L2 learners and bilinguals may experience interference from the non-target language (e.g., Yavas and Someillan, 2005). For example, while word-initial s+ consonant clusters are legal in English, a phonotactic constraint for Spanish is that s+ consonant clusters cannot exist at word onsets and an epenthetic /e/ (i.e., the addition of a vowel) must be added to render the word acceptable in Spanish. This incongruence between phonotactic constraints in the L1 and L2 might result in Spanish-like pronunciations and perceptions of English words during spoken word production and comprehension (e.g., *stable*, Spanish: *estable*).

### Comprehension

During receptive language processing, Spanish-only speakers have been shown to activate the epenthetic /e/ when viewing real Spanish words, even when the /e/ is removed from the word onset. Spanish speakers who performed a visual lexical decision task on words containing as+ and es+ consonant onsets showed facilitation of the epenthetic /e/ when primed with a Spanish word that had the /e/ onset removed (e.g., Spanish *stable*/“*estable*”; Hallé et al., 2008). The Spanish monolinguals in Hallé et al.’s (2008) study likely activated the epenthesis onset because Spanish was *overtly* presented and participants were judging lexicality in Spanish (not English).

Within-language activation of phonotactic constraints has been observed with monolinguals in other consonant–vowel contexts. For example, Japanese monolinguals applied an epenthesis constraint by adding a vowel (e.g., /u/) to an illegal consonant cluster in the coda of syllables when hearing Japanese-like non-words (e.g., they heard ‘mikdo,’ but perceived it as ‘mikudo’; Dupoux et al., 2001; Carlson et al., 2015). Hallé et al. (2008) discuss the process of epenthesis within consonant clusters as phonological repair (i.e., modifying auditory input that is phonologically illegal to conform to native language rules). Moreover, Parlato-Oliveira et al. (2010) examined how bilingual experience influenced the way the epenthesis constraint was repaired. Native Japanese-speaking adults who had been exposed to Portuguese (L2) when entering school demonstrated similar epenthesis patterns as native Portuguese listeners when processing illegal consonant clusters. Moreover, simultaneous Japanese-Brazilian bilinguals who were exposed to both languages from birth also demonstrated epenthesis repair similar to that observed in native Brazilian speakers (adding an /i/). Thus, previous results suggest that monolinguals and bilinguals potentially access and repair auditory input to align with their native or more proficient language.

While cross-linguistic activation of phonotactic constraints has yet to be established in comprehension, parallel language activation has been identified in other areas of phonology. Studies suggest that non-native listeners may rely on phonological categories from the non-target L1 during L2 auditory comprehension. For example, the two distinct vowels /𝜀/ and /æ/ are contrastive phonemes in English, but are non-contrastive allophones in Dutch. Consequently, Dutch learners of English, but not English monolinguals, erroneously activated ‘*deaf’* when primed with ‘*daf’* (Broersma and Cutler, 2011). If the highly proficient Dutch-English bilinguals tested in this study had mastered the /𝜀/ and /æ/ phonological category distinction of their L2 (English), then the findings would suggest access of L1 phonological categories during L2 processing. Alternatively, it is possible that even proficient L2 learners routinely rely on L1 categories during phonological processing in L2. Thus, previous research indicates that individuals are attuned to the phonotactic constraints of their L1 during native-language listening tasks (Hallé et al., 2008), and that bilinguals may potentially activate L1 phonological categories during L2 comprehension (e.g., Broersma and Cutler, 2011; Darcy et al., 2015). In the current study, we ask if bilinguals are also attuned to the phonotactic epenthesis constraint of the L1 (Spanish) during L2 (English) comprehension.

### Production

Evidence from word production also suggests that bilinguals are susceptible to cross-linguistic activation of phonological structures. Fabra and Romero (2012) found that L1 Catalan speakers of English produced English words with vowels (/i/, /𝜀/, /a/, /Λ/) that were less peripheral (i.e., sounded more like Catalan vowel phonology), than native English monolinguals. The less peripheral vowel effect disappeared as proficiency in English increased. Notably, all of the vowels except /Λ/ are shared across English and Catalan, thus the results suggest access of L1 phonological categories. As in comprehension (Broersma and Cutler, 2011), spillover effects of L1 phonological *categories* into L2 productions have been identified; but would there also be a similar effect with bilinguals accessing *phonotactic constraints* from the non-target language? Native Spanish speakers speaking English may at times produce words such as *estrict* in English (“strict”), adding an additional /e/ to the onset of words (Yavas and Someillan, 2005; see Roelofs and Verhoef, 2006, for review of bilingual cross-linguistic phonological access during production). While we have seen evidence for irrelevant-language phonological category and phonotactic constraint access during *production*, it is not clear whether bilinguals also access cross-linguistic phonotactic constraints during comprehension.

Previous investigations have explored the contexts in which cross-linguistic phonological activation could be facilitated. For example, cognates, which are words that overlap in form and meaning across languages (e.g., English: *stable/*Spanish: *estable*), have been used to test phonological co-activation during production (e.g., Amengual, 2012) and comprehension (e.g., Blumenfeld and Marian, 2007). It has been hypothesized that joint activation of similar-sounding translation equivalents enhances activation of phonological representations across languages. Amengual (2012) examined voice onset times (VOTs) of cognates and non-cognates produced by Spanish–English bilinguals. The results suggest that bilinguals produced longer (more English-like) VOTs on Spanish voiceless stops when producing cognates (e.g., English/Spanish *tumor*). In the presence of cognates, bilinguals may thus be more likely to experience activation of the non-target language. In an eye tracking study, English-German bilinguals’ looks to pictures representing cognate targets and cross-linguistic competitors suggested that cognates increased phonological co-activation of a less proficient non-target L2 during auditory word comprehension (Blumenfeld and Marian, 2007). It is possible that activation of cross-linguistic phonotactic constraints may become enhanced when phonological representations of the other language are co-activated. Including cognates in the current study provides a condition in which phonological co-activation of languages is most likely to occur.

The large body of research on parallel language activation in bilinguals, including phonological co-activation, has been captured by current models of bilingual language comprehension and production (e.g., Dijkstra and van Heuven, 2002; Shook and Marian, 2013). While current models of bilingual language *comprehension* do not specifically account for phonotactic constraints, one model of bilingual language *production*, the WEAVER++ model, does indeed propose that bilinguals access non-target language phonology (Roelofs and Verhoef, 2006). During bilingual production, activation of non-target language phonotactic constraints is thought to occur between encoding of the phonological word form for production and its phonetic realization. WEAVER++ posits that non-target language phonological representations and/or phonotactic constraints may intrude during encoding of words for production, and may combine with the phonological representations or phonotactic constraints of the target language to affect phonetic realization (e.g., applying the Spanish epenthetic /e/ to an English s+ consonant cluster, *estudy*).

In summary, while current experimental and theoretical work on bilingual language comprehension suggests that bilinguals co-activate phonological representations of the non-target language, it remains unclear whether they access cross-linguistic phonotactic constraints during language comprehension. The current study has the potential to expand upon the existing knowledge base for the types of cross-linguistic phonological interactions that occur during bilingual language comprehension.

### Current Study

In the current study, we explore for the first time whether bilinguals co-activate phonotactic constraints from the non-target language during comprehension. Furthermore, while phonotactic constraint activation has been observed empirically during production (e.g., Yavas and Someillan, 2005), we test whether bilinguals also access phonotactic constraints during comprehension. Thus, the current study attempts to provide evidence for the extent to which cross-linguistic structures are accessed during language comprehension in bilinguals.

In order to measure if bilinguals activated phonotactic constraints in the non-target language (Spanish), we employed a cross-modal phonological priming lexical decision (PPLD) task. We used cognates and non-cognates to index availability of phonotactic constraints in different contexts of cross-linguistic phonological activation (e.g., Van Hell and Dijkstra, 2002; Blumenfeld and Marian, 2007). For example, when Spanish–English bilinguals hear the cognate *stable* unfold through the acoustic stream, they may initially activate phonological cohorts from both languages (e.g., *stand*, *stain*, *sink/sárten*, e.g., Blumenfeld and Marian, 2007, 2013) and the Spanish translation equivalent (i.e., *estable*; e.g., Linck et al., 2009). Critically, when hearing ‘*stable*,’ they may also activate phonological cohorts that overlap with Spanish through phonotactic constraints and phonological form (e.g., *estándar*/standard) and potentially even cohorts that overlap with Spanish through phonotactic constraints only (e.g., *edad*/age). As an alternative to activation of phonological and phonotactic cohorts upon hearing ‘stable’ in English, native Spanish speakers may perceptually repair ‘stable’ to “e-stable,” (/esterbәl/) and therefore may not hear ‘stable’ (Hallé et al., 2008). Whether bilinguals access neighbors containing phonotactic constraints through spreading activation and mediated priming (English ‘stable’ activates Spanish /e/ onset words) or repair the auditory input to have the epenthesis onset, both scenarios suggest that bilinguals may access the phonotactic constraint of /e/ onset from their L1 and apply it during L2 processing.

**FIGURE 1:**
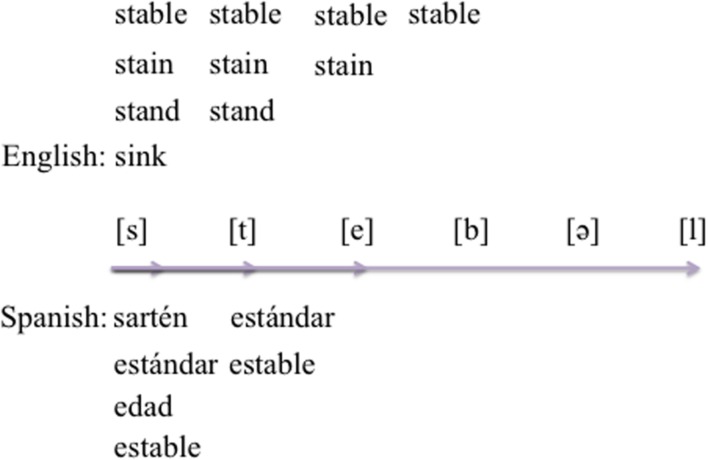
**Example of competitor activation with an English–Spanish cognate (*stable*) tor bilinguals**. As the word unfolds through time, bilinguals may access multiple phonological cohorts across languages until the acoustic stream matches the target word representation. In the present study, words like *stable* will serve as auditory primes. Words such as *especie* represent phonological-form as well as phonotactic-constraint overlap between English and Spanish, while words such as *edad* represent phonotactic -constraint-only overlap between English and Spanish.

Here, we examine both phonotactic-constraint-*and*-form access as well as phonotactic-constraint-*only* access across English and Spanish in order to dissociate constraint from form overlap (e.g., *edad* and *estándar*, respectively, see **Figure 1**). We will henceforth refer to the phonotactic-constraint-*and*-form manipulation as the PCF condition, and to the phonotactic-constraint-*only* manipulation as the PC condition. We focused on the Spanish epenthesis constraint (/e/ onset, e.g., English ‘*estudy*’) because it is a commonly observed phenomenon that occurs in production with native Spanish speakers speaking English, and thus presents a good starting point in exploring a phonotactic constraint during comprehension. The Spanish epenthesis constraint is particularly suitable to the current experimental manipulation because of its potential to be primed with English words that violate the Spanish phonotactic constraint.

We hypothesized that Spanish–English bilinguals would access Spanish (L1) phonotactic constraints during English (L2) comprehension. The goal was to examine the presence or absence of non-target language phonotactic constraint activation when phonological and lexico-semantic (cognate) or no (non-cognate) overlap was present between auditory primes and their translation equivalents. Moreover, we predicted that when bilinguals were primed with an /st/ or /sp/ word, they would access shared phonological (e.g., ‘strong’/*stand/estándar*), lexical (e.g., ‘strong’/*fuerte*), and potentially phonotactic constraint (e.g., ‘strong’/*edad*) neighbors across languages. Presentation of visual /est/, /esp/, or /e/ non-word targets (e.g., *esteriors*) would then limit cross-linguistic activation to strictly phonological forms (/es/ onset) and/or phonotactic constraints (/e/ onset) that had been previously activated by the prime (e.g., Dijkstra and van Heuven, 2002; Shook and Marian, 2013). Restricted activation of phonological representations (/e/ and /es/ onsets) across primes and targets would in turn facilitate lexical selection, and thus yield faster reaction times when making a lexical decision. Given that the phonology of critical targets (e.g., *esteriors*) was expected to activate partial phonological form and phonotactic constraints of Spanish, but no specific Spanish lexical items, we predicted that there would be no lexical interference from Spanish. These predictions are supported by previous research using a lexical decision task and manipulating the amount of word-initial phoneme overlap across languages (e.g., no-overlap, 1-phoneme overlap, 2 phoneme-overlap, and 3-phoneme overlap). When Russian-English bilinguals processed words in the non-native language (English), cross-linguistic phonological overlap of word onsets was associated with faster reaction times as compared to no phonological overlap (Marian et al., 2008). In the current study, we expected that s+ consonant priming would restrict activation to words with /e/ and /es/ onsets. Therefore, Spanish–English bilinguals would be able to quickly search through a constrained space within the lexicon of s+ consonant, es+ consonant, and e+ consonant onset words to make a lexical decision. In contrast, for control non-words that did not conform to the epenthesis constraint, phonological representations would need to be activated for the first time, delaying the subsequent lexical search, and resulting in slower reaction times.

Including the cognate and non-cognate priming conditions, as well as the target conditions with PCF and PC overlap, ensured that bilingual participants would experience local (i.e., intermittent) co-activation of Spanish throughout the task. We predicted that cognates (e.g., *stable* /sterbәl */estable* /estaβle/) would facilitate activation of Spanish translation equivalents more strongly than non-cognates (e.g., *strong/fuerte*) based on phonological form overlap (e.g., *stable* /sterbәl/ *estable* /estaβle/). Following the /sp/ and /st/ primes, PCF non-word targets that overlapped with Spanish /esp/ or /est/ onsets would potentially activate Spanish phonological form in addition to the constraint. The PC targets shared just the Spanish /e/ onset (epenthesis constraint), therefore activating Spanish to a lesser degree. We expected that, if bilingual participants would locally co-activate Spanish, effects on /e/ and /es/ non-word targets would be present only when directly preceded by /sp/ or /st/ primes, but not when preceded by control primes (e.g., workers).

We specifically predicted, across conditions on the PPLD task, that if cognate auditory primes (e.g., *stable*) facilitated non-target language phonotactic constraint and phonological form access, then bilinguals would demonstrate faster reaction times to visual letter strings that contained the previously-activated phonological cohorts (e.g., PCF non-words: *esteriors*), as compared to conditions in which less or no phonological overlap was present (e.g., controls: *stable/hereander* or *workers/hainsail*). In addition, we expected that if the non-cognate auditory primes (e.g., *strong*) facilitated phonotactic constraint access, then the bilingual group would demonstrate faster reaction times to non-word targets with PCF overlap (e.g., *estimagle*), relative to control trials (e.g., *strong/atongside*). However, this facilitation effect was predicted to be less strong than the cognate prime/PCF trials because of the absence of overlap between translation equivalents in the non-cognate prime. If bilinguals routinely activated phonotactic constraints across their two languages, then we would also expect to see similar reaction time facilitation effects for non-word targets that overlapped only with the phonotactic constraint when paired with cognate and non-cognate primes (e.g., /e/-only onset: *stable/elopevent* and *strong/encimpass*, respectively). We expected that this facilitation effect would be less robust in comparison to the PCF overlap condition, since phonological form overlap was not present. We included a control-prime condition, which was not expected to activate Spanish due to either phonotactic constraint or lexico-semantic overlap, as no overt overlap between English and Spanish was present in the control stimuli.

## Materials and Methods

### Participants

Participants included 22 Spanish–English bilinguals and 23 English monolinguals, ages 18–33. Monolinguals and bilinguals were recruited via word-of-mouth, e-mails to local student and community organizations, flyers posted around campus and the community, as well as through existing participant databases. This study was carried out in accordance with the recommendations of Northwestern University’s Institutional Review Board with written informed consent from all subjects. All subjects gave written informed consent in accordance with the Declaration of Helsinki. Any of the monolingual English participants who had a self-reported Spanish speaking proficiency of greater than 3 (0–10 scale) on the language experience and proficiency questionnaire (LEAP-Q; Marian et al., 2007) did not participate in the experiment. Bilinguals were native Spanish speakers, were exposed to Spanish at least 30% of the time daily, and acquired English at age 5 or later. See **Table 1** for additional participant information. Monolinguals and bilinguals differed on English age of acquisition (*p* < 0.001), current exposure to English (*p* < 0.001), and foreign accent in English (*p* < 0.01). Participants were matched on age, non-verbal cognitive reasoning (WASI; PsychCorp, 1999), and working memory (backward digit span; Woodcock et al., 2001/2007).

**Table 1:** Linguistic and cognitive background of Spanish–English bilingual (*n* = 22) and English monolingual (*n* = 23) participants.

	Bilinguals mean (*SE*)	Monolinguals mean (*SE*)
Age	24.09 (0.84)	22.95 (0.74)
Age of Spanish acquisition	0.45 (0.12)	–
Age of English acquisition^∗∗^	6.05 (0.49)	0.18 (0.08)
Current exposure to Spanish	36.77% (6.40)	–
Current exposure to English^∗∗^	62.50% (6.80)	98.65% (0.69)
Foreign accent in Spanish (0–10 scale)	2.10 (0.44)	–
Foreign accent in English (0–10 scale)^∗^	2.82 (0.56)	0.73 (0.56)
Spanish receptive vocabulary (NIH Toolbox)	116.77 (2.84)	–
English receptive vocabulary (NIH Toolbox)	110.14 (3.55)	118.86 (3.39)
Self-reported Spanish proficiency (0–10 scale)	9.03 (0.14)	–
Self-reported English proficiency (0–10 scale)	8.95 (1.10)	9.83 (0.05)
WASI, matrix reasoning	29.27 (0.53)	28.78 (0.61)
Backward digit span	7.33 (1.20)	10.14 (1.10)

*^∗∗^p < 0.001; ^∗^p < 0.01.*

### Materials

The English cross-modal PPLD task was designed to measure cross-linguistic activation of the Spanish phonotactic constraint (the epenthetic /e/) in the presence of phonological and lexico-semantic overlap between languages (cognate auditory primes) or in the absence of phonological overlap between languages (non-cognate auditory primes) through accuracy and reaction time to target identification. The within-subjects independent variables included prime type (cognate, non-cognate, control) and target type (PCF overlap non-word, PC non-word, non-word control, word control). The /st/ and /sp/ consonant clusters were chosen because they are illegal consonant clusters in Spanish without the obligatory epenthetic /e/ at the word onset. In addition, the two consonant clusters are present in a sufficient number of English cognates and non-cognates to generate stimuli for the current study.

The cross-modal PPLD task was programmed in MatLab (Psychtoolbox add-on; Brainard, 1997; Pelli, 1997; Kleiner et al., 2007). The auditory primes were recorded in a soundproof room (44,100 Hz, 16 bits) by a native female speaker of English. The audio recording was split into individual audio files and all files were normalized (via audio compression) in Praat (Boersma and Weenink, 2013) and exported into MatLab (Brainard, 1997; Pelli, 1997; Kleiner et al., 2007). Each prime type was paired with each target type (3x4), resulting in 12 different pairing combinations and the repetition of each prime four times and each target three times throughout the duration of the experiment. **Table 2** depicts examples of stimulus pairings for each prime and target type.

**Table 2:** Example stimulus pairings and total number of each item type.

Auditory prime	Phonotactic constraint + Phonological form target	Phonotactic constraint only target	Non-word control target	Word control target
30 Cognates *(stable)*	30 *(esteriors)*	30 *(elopevent)*	30 *(hereander)*	30 *(flattened)*
30 Non-cognates *(strong)*	30 *(estimagle)*	30 *(encimpass)*	30 *(atongside)*	30 *(daughters)*
30 Controls *(workers)*	30 *(esported)*	30 *(ebvision)*	30 *(hainsail)*	30 *(kneeling)*

A total of 360 critical trial pairs were created, comprised of cognate primes (30 items), non-cognate primes (30 items), and control primes (30 items). Each of the auditory primes was paired with a visual target that included non-words that overlapped with Spanish via phonotactic constraint and phonological form (/es/ onset, 30 items), via phonotactic constraint only (/e/ onset, 30 items), non-words that did not overlap with Spanish via phonotactic constraint or form (non-word control, 30 items), or a real word in English that did not overlap with Spanish (word control, 30 items). The PCF (/es/ onset) non-word targets were controlled in such a way that they overlapped cross-linguistically with only the first three letters of the Spanish translation of the cognate prime [e.g., cognate prime *stable* (***est****able*) was paired with /es/ non-word target (***est****eriors*)]. Controlling the targets in this manner would avoid any priming effects due to additional phonological and orthographic overlap. The PC non-word targets overlapped with the cognate prime’s translation equivalent only at the /e/ onset [e.g., cognate prime *stable* (***e****stable*) was paired with /e/ non-word target ***e****lopevent*)]. To a) balance the proportion of word (50%) versus non-word (50%) trials, and b) prevent the participants from noticing any patterns concerning the critical stimulus pairs, 45 auditory prime fillers and 45 visual target fillers (180 total trial pairs) were also generated. Twelve additional pairs were created as practice trials. The experiment was divided into four blocks and the items were pseudo-randomized such that no two consecutive trials contained cognate primes. Consistent with cross-linguistic priming studies employing lexical decision tasks, cognate and non-cognate trials were presented in an intermixed order (Duyck et al., 2007; Siyambalapitiya et al., 2009; Davis et al., 2010; Dijkstra et al., 2010). Finally, trial order was counterbalanced (reversed) across participants.

All stimuli were controlled for various lexical characteristics. The three types of auditory primes did not differ on any of the lexical characteristics listed in Appendix A (all *p*s > 0.05).

The four types of lexical decision targets also did not differ on any of the lexical characteristics (*p*s > 0.05), with the exception of lexical decision reaction time (LDT RT) and lexical decision z-score (LDT Zscore) in which non-words had slower lexical decision response times than words in the normed sample, *p*s < 0.05 (Balota et al., 2007). See Appendix B for means and standard deviations. Similar to previous studies (e.g., Martín et al., 2010), we did not control for part of speech (auditory primes) due to the number of lexical characteristics on which the stimuli needed to be matched.

### Procedure

Tasks were administered in the following order:

(1) the LEAP-Q (Marian et al., 2007) to obtain linguistic background information and current language exposure, and to ensure that each participant met the criteria for the study;
(2) the cross-modal PPLD task (auditory prime, visual target) to examine cross-linguistic phonotactic constraint access;
(3) a non-linguistic Stroop task to index competition resolution abilities (adapted from Blumenfeld and Marian, 2014);
(4) a backward digit span task (numbers reversed, Woodcock et al., 2001/2007) to index working memory;
(5) the Wechsler Abbreviated Scale of Intelligence (WASI; PsychCorp, 1999) to index non-verbal cognitive reasoning; and
(6) the NIH Cognition Toolbox Battery (National Institutes of Health Toolbox Cognition Battery (NIH Toolbox CB), 2013) picture vocabulary test, as a measure of English (bilinguals and monolinguals) and Spanish (bilinguals only) proficiency.

**FIGURE 2:**
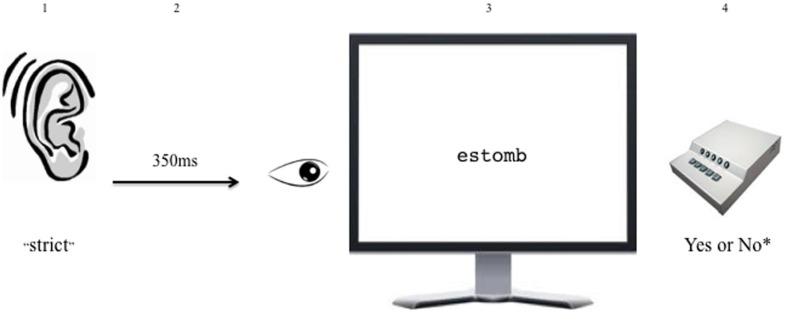
**Example trial from cross-modal phonological priming lexical decision task**. In this example, participants hear the English–Spanish cognate auditory prime *strict* and 350 ms after the offset of the prime, view the phonotactic-constraint-and-form overlap non-word visual target *estomb*, on which they perform a lexical decision (^∗^Yes response = English real word, No response = non-word).

Participants were seated in a quiet room with a single iMac computer and were asked to pay attention to the word they heard and then respond by indicating whether what they saw on the screen was a word or non-word in English as quickly and as accurately as possible. After the instructions and 12 practice trials, participants performed the experimental task in which they first heard an auditory prime (cognate, non-cognate, control, filler) and then saw a visual written target (PCF overlap non-word, PC non-word, non-word control, word control, filler) on the screen after a 350 ms inter-stimulus interval (ISI). During presentation of the auditory prime through the 350 ms ISI, participants viewed a central fixation crosshair on the computer screen. Previous studies using similar priming techniques have shown effects of parallel activation 350–500 ms post-stimulus onset (e.g., Martín et al., 2010; Blumenfeld and Marian, 2013). The visual targets were presented in the center of a white screen in black, size 16 font, Courier, and the left/right shift keys represent yes/no responses. Presentation of written words lasted until the participant made a response or for 3,000 ms after the onset of the display (see **Figure 2**).

Participants were given three short, but untimed, breaks in between each of the four blocks. The total time to complete this task was approximately 30 min. Participants performed the remaining tasks, then were debriefed about the study and compensated. The total study duration was approximately 2 h.

### Coding and Analysis

For the PPLD task, reaction times and accuracy rates were analyzed. Reaction times were measured from the onset of the visual lexical decision target (PPLD task). Filler trials were not analyzed, as they only served to balance the word/non-word ratio. Incorrect trials and trials 2.5 standard deviations above and below the mean reaction time were disregarded for both tasks. Means and standard deviations for each condition (12 critical conditions) were then calculated.

## Results

### Overall Accuracy Effects on the PPLD Task

We examined lexical decision accuracy, expecting that decisions on non-words would be less accurate than on real words based on previous research (de Groot et al., 2000). A 3 (auditory prime: cognate, non-cognate, control) × 4 (visual target: PCF overlap non-word, PC non-word, non-word control, word control) × 2 (language group: monolingual, bilingual) repeated measures ANOVA was conducted on the lexical decision targets. There was a main effect of target, *F*(3,129) = 4.26, *p* < 0.01, $\eta_{p}^{2}$ = 0.09, with Bonferroni-corrected pairwise *post hoc* comparisons revealing that participants were more accurate on PCF overlap non-word trials (e.g., *esteriors*; *M* = 96.89%, *SE* = 0.47) than on word control trials (e.g., *flattened*; *M* = 94.87%, *SE* = 0.90), *p* = 0.045. While we did not anticipate higher accuracy for non-words, we reason that this accuracy effect may have been due to participants using more time to make a decision on non-words than on words, as evidenced by increased reaction times for non-words (see below).

### Overall Reaction Time Effects on the PPLD Task

We next examined whether monolinguals would be faster overall in their lexical decision response rates than bilinguals, as bilinguals were performing a lexical decision in their L2 (Dijkstra et al., 1999). Further, we tested whether participants were slower to respond to non-words than words, a pattern demonstrated in previous research (Dijkstra et al., 1999). There was a main effect of language group, *F*(1,43) = 11.70, *p* < 0.01, $\eta_{p}^{2}$ = 0.21, indicating that monolinguals (*M* = 655.96 ms, *SE* = 46.10) indeed responded to targets more quickly than bilinguals (*M* = 881.31 ms, *SE* = 47.10), *p* < 0.01. A main effect of visual target condition was also identified, *F*(3,129) = 16.02, *p* < 0.001, $\eta_{p}^{2}$ = 0.27, with Bonferroni-corrected pairwise *post hoc* comparisons indicating the following patterns: participants were faster to respond to PCF overlap non-word trials (e.g., *esteriors*; *M* = 758.99 ms, *SE* = 31.49) than to non-word controls (e.g., *hereander*; *M* = 800.52 ms, *SE* = 37.11), *p* < 0.001; faster to respond to PC non-word trials (e.g., *elopevent*; *M* = 770.47 ms, *SE* = 32.54) than to non-word controls (e.g., *hereander*; *M* = 800.52 ms, *SE* = 37.11), *p* < 0.01; faster to respond to word-control trials (e.g., *flattened*; *M* = 744.50 ms, *SE* = 31.90) than to PC non-word trials (e.g., *elopevent*; *M* = 770.47 ms, *SE* = 32.54), *p* < 0.05; and faster to respond to word-control trials (e.g., *flattened*; *M* = 744.50 ms, *SE* = 31.90) than to non-word-control trials with other word onsets (e.g., *hereander*; *M* = 800.52 ms, *SE* = 37.11), *p* < 0.001. Thus, reaction time differences across target conditions confirmed faster overall responses in monolinguals than bilinguals and faster responses to words over non-words. Effects of target condition warranted further follow-up analyses across monolinguals and bilinguals.

### Monolingual versus Bilingual Reaction Time Performance

Next, related to our prediction of greater cross-linguistic activation effects in bilinguals than monolinguals, we examined whether differences in performance across target conditions would be greater for bilinguals than monolinguals. Indeed, an interaction emerged for reaction times between target type and language group, *F*(3,129) = 4.18, *p* < 0.01, $\eta_{p}^{2}$ = 0.09. Bonferroni-corrected pairwise comparisons revealed that, relative to monolinguals, bilinguals showed additional reaction time effects across target conditions, with faster reaction times to PCF overlap non-words (*M* = 866.49 ms, *SE* = 59.16) than to non-word control trials (*M* = 928.81 ms, *SE* = 68.28), *p* < 0.01, and a marginal effect of faster reaction times to PC non-word trials (*M* = 885.55 ms, *SE* = 60.30) than to non-word control trials (*M* = 928.81 ms, *SE* = 68.28), *p* = 0.058. Monolinguals did not demonstrate such effects, *p*s > 0.05.

### Phonotactic Constraint Activation between Cognate and Non-cognate Primes and Target Conditions

**FIGURE 3:**
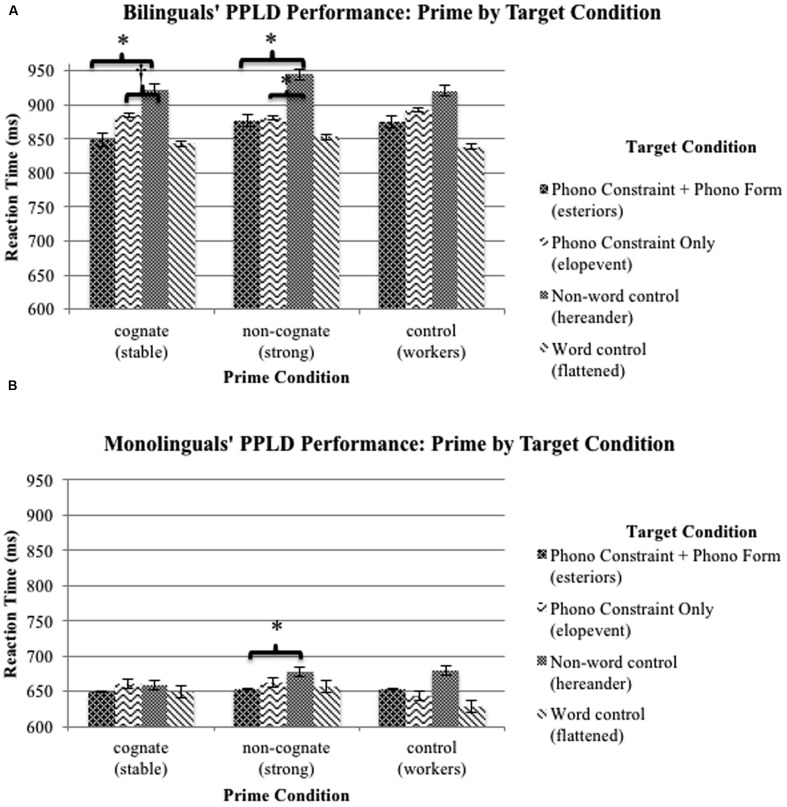
**Reaction times (RTs) on the cross-modal PPLD task for bilinguals **(A)** and monolinguals **(B)** by condition**. Error bars = standard error. Differences marked for primary conditions of interest: ^∗^*p* < 0.05; ^†^*p* = 0.082.

Finally, we tested our key prediction following the hypothesis of bilinguals’ activation of irrelevant-language phonotactic constraints during comprehension. We conducted planned follow-up *t*-test comparisons within monolingual and bilingual groups to probe for reaction time effects across prime and target conditions of interest. It was expected that some priming effects would occur for monolinguals, as there was /st/ or /sp/ overlap between the prime and target. Indeed, a significant difference was observed for monolinguals, with faster reaction times to PCF overlap targets (e.g., *estimagle*) preceded by non-cognate primes (e.g., *strong*; *M* = 662.62 ms, *SE* = 27.10) than to non-word controls (e.g., *atongside*) preceded by non-cognate primes (*M* = 677.61 ms, *SE* = 32.10), *t*(22) = −2.51, *p* = 0.02. However, bilinguals demonstrated several significant reaction time differences across prime and target conditions in line with non-target language phonotactic constraint activation. Bilinguals were faster to respond to PCF overlap non-word trials (e.g., *esteriors*) preceded by cognate primes (e.g., *stable*; *M* = 848.45 ms, *SE* = 57.70) than to non-word controls (e.g., *hereander*) preceded by cognate primes (*M* = 922.29 ms, *SE* = 66.42), *t*(21) = –3.94, *p* = 0.001. Bilinguals were also marginally faster to respond to PC non-word trials (e.g., *elopevent*) preceded by cognate primes (*M* = 883.83 ms, *SE* = 56.68) than to non-word control trials preceded by cognate primes (*M* = 922.29 ms, *SE* = 66.42), *t*(21) = –1.83, *p* = 0.082. Finally, bilinguals were faster to respond to PCF overlap non-word targets (e.g., *estimagle*) *and* PC non-word targets (e.g., *encimpass*) preceded by non-cognate primes (e.g., *strong*; *M* = 876.33 ms, *SE* = 61.85; *M* = 881.14 ms, *SE* = 62.67, respectively) than to non-word controls preceded by non-cognate primes (M = 944.39 ms, SE = 72.11), *t*(21) = –4.63, *p* < 0.001; *t*(21) = –3.56, *p* < 0.01, respectively. (See **Figures 3A,B** for the bilingual versus monolingual reaction time by condition comparison.)

The results within the bilingual group demonstrate significant effects of Spanish phonotactic constraint activation during English comprehension. Bilinguals demonstrated faster reaction times, relative to control conditions, to PCF overlap non-words when primed with cognates, as well as faster reaction times to PCF overlap non-words and PC overlap non-words when primed with non-cognates.

## Discussion

Our goal was to explore whether bilinguals accessed phonotactic constraints from the irrelevant language (Spanish) during English-only receptive language processing. Participants heard English words that were chosen to enhance cross-linguistic phonological activation (cognates: *stable*), that did not provide cross-linguistic phonological activation beyond the shared word onset (non-cognates: *strong*), or that were non-facilitatory of Spanish /es/ or /e/ words (controls: *workers*). Immediately after hearing the auditory prime, participants performed a lexical decision on either (1) an English-like non-word that corresponded to Spanish via phonotactic constraint (epenthesis, /e/) and form (/s/) overlap (/es/ non-words: *esteriors*), (2) PC overlap (/e/ non-words: *elopevent*), (3) on an English-like non-word that did not correspond to Spanish phonotactic constraints or form (non-word controls: *hereander*), (4) or on a real-word control (*flattened*). Both monolinguals’ and bilinguals’ performance patterns were consistent with co-activation of phonologically similar representations. That is, both monolinguals and bilinguals showed facilitated responses to constraint-and-form overlap non-words. However, bilinguals displayed patterns of parallel language activation based on phonological form and/or constraint overlap, as demonstrated by significant reaction time differences to PCF overlap non-words when primed by both cognates and non-cognates and PC overlap non-words when primed with non-cognates compared to control conditions. See **Table 3** for a summary of results.

**Table 3:** Summary of results for bilinguals and monolinguals.

	Cognate prime Phonotactic constraint + form target: *stable/esteriors*	Cognate prime Phonotactic constraint only target: *stable/elopevent*	Non-cognate prime Phonotactic constraint + form target: *strong/estimagle*	Non-cognate prime Phonotactic constraint only target: *strong/encimpass*
Bilinguals	✓	†	✓	✓
Monolinguals	–	–	✓	–

*† = Marginally significant difference, ✓ = Significant difference. Conditions of interest compared to the non-word control condition.*

### Non-target Language Phonotactic Constraint Access via Non-cognates

We aimed to tease apart PCF access in the presence (cognate primes) and absence (non-cognate primes) of previous cross-linguistic activation. With monolinguals, we expected to see a small amount of priming, as there was English phonological overlap between the prime and target conditions of interest. Critically, bilinguals but not monolinguals were found to activate the Spanish epenthesis constraint with PCF and PC overlap non-word targets when primed with English non-cognate words that had s+ phonology onsets. This finding suggests that proficient Spanish–English bilinguals may activate phonotactic constraints from their L1 when listening to English words.

### Non-target Language Phonotactic Constraint Access via Cognates

There were no significant differences across the cognate prime and non-word target conditions for monolinguals. Bilinguals, however, appeared to have accessed the Spanish phonotactic constraint when primed with cognates, but that access was limited to PCF overlap non-word trials; the effect for PC overlap non-word trials was only marginally significant. This finding is consistent with previous results of bilingual parallel language activation in the presence of cognate words (e.g., Blumenfeld and Marian, 2007; Shook and Marian, 2013). Yet contrary to previous findings and expectations (e.g., Blumenfeld and Marian, 2007; Shook and Marian, 2013), cognates were found to facilitate cross-linguistic access to phonotactic constraints to a lesser extent than did non-cognates. The finding that non-cognates independently activated bilinguals’ Spanish via phonotactic constraint and phonological form overlap suggests that lexico-semantic activation of the non-target language (via cognate primes) is not needed to facilitate Spanish phonotactic constraints. Instead, phonological form overlap alone (via non-cognate primes) may consistently activate Spanish phonotactic constraints.

Taken together, the current findings suggest that Spanish–English bilinguals may activate a phonological epenthesis constraint in the non-target language (e.g., the constraint of adding an /e/ to the onset of an s+ consonant cluster) during comprehension when primed by *non-cognates*, with smaller but similar effects for *cognates*. This finding is at odds with initial predictions that a phonotactic constraint activation effect would be stronger with cognate primes, as cognate processing yields broader activation of the lexico-semantic and phonological system across both languages (Dijkstra and van Heuven, 2002; Shook and Marian, 2013). However, preliminary conclusions can be drawn from the current findings based on the cognate and non-cognate differences we observed. While it is believed that cognates, compared to non-cognates, increase co-activation of the two languages, bilinguals may need to work harder to protect from cross-linguistic competition resulting from cognates. In the current study, enhanced parallel language activation may result in an increased likelihood of intrusion from non-target language phonotactic constraints. For example, when a bilingual makes a decision on whether a string of letters forms a word, or when s/he produces a word when cross-linguistic competition (i.e., cognates) is present, s/he may emphasize language-specific plans in her response to help resolve competition. Consistently, Nip and Blumenfeld (2015) found that production of cognate sentences was associated with a greater range of speech articulator movements than non-cognate sentences in the L1 of L2 learners. Greater ranges of movement have been associated with more detailed phonological specification (Lindblom, 1990), suggesting more care in the precise articulation of the target language. Thus, across both comprehension and production, the presence of cognates may necessitate muting of phonotactic constraints from the non-target language so that bilinguals can use language-specific plans. With non-cognates, such muting is not necessary, likely due to decreased amounts of cross-linguistic competition. This preliminary conclusion is in line with the prediction that more cognitive resources may be required to inhibit the non-target language during cognate word processing (Green, 1998).

### Implications for Current Accounts of Parallel Activation

The findings from this study suggest parallel activation of phonotactic constraints across two languages and are thus consistent with previous research demonstrating parallel activation of phonological (Marian and Spivey, 2003; Blumenfeld and Marian, 2007, 2013; Mercier et al., 2014) and lexico-semantic (e.g., Martín et al., 2010) cohorts in bilinguals during auditory and visual word processing. The current study adds to the existing bilingual language comprehension literature an additional level within cross-linguistic phonological access, the phonotactic constraint. As such, this study complements bilingual language production research that suggests bilinguals access phonotactic constraints from the non-target language (e.g., Yavas and Someillan, 2005). Furthermore, these results highlight the additional linguistic competition that bilinguals manage, relative to monolinguals, during language processing: while monolinguals demonstrated minimal interference between the primes and targets across conditions, suggesting activation of phonological representations within-language, bilinguals experienced activation from the non-target language, at the levels of both phonotactic constraint and phonological form competition.

Moreover, using the existing framework from models of bilingual language comprehension, we can extend current explanations of parallel language activation in bilinguals to incorporate the findings of the current project. Two models of bilingual language comprehension, the Bilingual Language Interaction Network for Comprehension of Speech model (BLINCS; Shook and Marian, 2013) and the Bilingual Interactive Activation + model (BIA+; Dijkstra and van Heuven, 2002), suggest that bilinguals activate both languages in parallel during single language comprehension. While both of these comprehension models do posit language co-activation based on phonology (e.g., English: *plug*, Spanish competitor: *pluma*, or *pen*), no specific claims are made about phonotactic constraint access of the non-target language.

Within the BLINCS model, bilinguals are thought to access both of their languages across various interconnected levels of processing, including phonological, phono-lexical, ortho-lexical, and semantic representations. The levels rely on a network of self-organizing maps, which provide an algorithm for learning. With activation of cross-linguistic phonological representations during comprehension, as auditory input unfolds through time, the input is first mapped onto the closest node that best matches the target (e.g., language co-activation of translation equivalents, English: *strong*/Spanish: *fuerte*), and the node is altered to become more similar to the input. Based on current findings, we can extend the BLINCS model by suggesting that nearby nodes, which include words that activate words consistent with non-target language phonotactic constraints (e.g., English: *strong*/Spanish: *edad*), might then be adapted to become more similar to the input. The space around the input, containing words following similar phonological patterns, becomes more uniform as the target word is selected. The BLINCS model also has the potential to explain the differences in processing observed across cognate and non-cognate prime conditions and non-target language phonotactic constraint access. It is possible that when bilinguals process cognates, neighboring words following the /e/ epenthesis constraint are more quickly activated than when processing non-cognates. Over time, the cognate neighbors are suppressed as the target word is reached for selection. When processing non-cognates that activate the /e/ epenthesis constraint, neighbors also become activated, however, target word selection may take longer due to the lack of lexico-semantic overlap. Thus, stronger effects of non-target language phonotactic constraint activation may emerge when processing non-cognates.

Like the BLINCS model, the BIA+ model of bilingual written word recognition (Dijkstra and van Heuven, 2002) supports language non-selectivity (integrated bilingual lexicon) and spreading activation of cross-linguistic phonological neighbors during bilingual language comprehension. The BIA+ model states that when orthographic representations become active, associated within- and between-language phonological representations start to become activated as well. However, the model does not account for how and if phonotactic constraints from the irrelevant language are accessed, which is what was observed in the current study. As non-target language phonotactic constraints become active, so too phonological neighbors may become active that include cohorts of both languages (e.g., English and Spanish). For example, English *strong* may activate an intermediate form where the epenthesis constraint is applied, *estrong*, which may in turn co-activate Spanish words that overlap in phonological form (e.g., *estar*/*edad*, English: to be/age). It is thus possible that phonotactic constraint cohort members from the irrelevant language may be activated during visual word processing in addition to non-target language orthographic, and phonological cohorts. Both the BIA+ and BLINCS models can be minimally extended to provide a theoretical framework to account for parallel activation of phonotactic constraints across languages in bilinguals.

### Limitations and Future Directions

The PCF overlap (/es/) non-words used in the current study could have facilitated global activation of Spanish throughout the entire task, as the non-words were Spanish-like in form. However, this was likely not the case since we provided an additional condition in which irrelevant-language phonotactic constraint access was possible, the PC overlap (/e/ non-words) condition. Including the two conditions allowed us to dissociate between phonotactic constraint and phonological form overlap with Spanish. Indeed, we found that when primed with non-cognates, bilinguals accessed the /e/ onset phonotactic constraint when making a lexical decision on the PC overlap targets. This effect was also marginally significant with cognate primes. Therefore, we can rule out that Spanish was activated only in the PCF condition, based on the evidence from the PC overlap condition. Relatedly, the finding that effects on /e/ and /es/ non-word targets were present only when directly preceded by an /sp/ and /st/ prime (and not control primes) suggests that there was no global activation of /e/ and /es/ phonology across the entire task. Finally, bilinguals, but not monolinguals, showed a significant effect for the PC condition when primed with non-cognates.

Future research is needed to further explore the possibility that Spanish–English bilinguals perceptually repair L2 auditory input i.e., primes such as stable) to have an /e/ onset, as has been shown on a Spanish-language task in Spanish monolinguals (Hallé et al., 2008). If bilinguals experienced a perceptual illusion of repairing the auditory prime to “e-stable” (/esterbәl/), this would also be suggestive of access to the phonotactic epenthesis constraint in the L1. While perceptual repair remains an alternative explanation to the current results, this alternative explanation is also consistent with the hypothesis of cross-linguistic activation. Thus, while the present study provides evidence that bilinguals access phonotactic rules from the non-target language during comprehension, whether the underlying mechanism(s) is constraint activation or perceptual repair remains an open question.

The contrast identified here between non-cognate and cognate words suggests that language selection mechanisms during phonotactic constraint competition also warrant further examination. For example, research might identify the time course of non-target language phonotactic constraint access (i.e., duration of L1 interference in an L2 context) during language comprehension, which will shed light on mechanisms involved with activation and suppression of non-target language phonotactic constraints. In addition, our findings showed effects of non-target language phonotactic constraint access with /es/ or /e/ onset non-word targets, not across actual English and Spanish words. We believe our results have clear implications for theoretical models of bilingual language comprehension, though stronger evidence for cross-linguistic activation of phonotactic constraints would be provided by a replication study using actual English and Spanish word targets. Moreover, varying the age of acquisition of the L2 (e.g., earlier than 5) will elucidate whether simultaneous versus sequential bilinguals experience phonotactic constraint access to a similar degree.

Finally, future studies may test different sets of language-specific phonotactic constraints to examine whether such constraints are generally accessible across languages. For example, Spanish does not permit consonant clusters at the end of words, and oftentimes native Spanish speakers reduce final consonant clusters when speaking English (e.g., *soun* for *sound*). As is the case in cross-linguistic co-activation of phonological representations (e.g., Marian and Spivey, 2003; Blumenfeld and Marian, 2007, 2013), it is possible that phonotactic constraints are especially likely to become co-activated across languages when they are specific to the dominant language. Furthermore, such constraints may become active cross-linguistically in contexts where the less dominant language violates a phonotactic constraint in the native language.

## Conclusion

To conclude, results from the current study demonstrate that Spanish–English bilinguals access Spanish phonotactic constraints during English *comprehension*. Moreover, bilinguals’ access to structures across both languages during spoken word comprehension is not limited specifically to phonology, but also applies to phonotactic constraints. Finally, the degree of phonological and semantic overlap across languages, as manipulated in cognate vs. non-cognate words, may modulate the extent to which cross-linguistic constraints are available, thus providing further support that the bilingual language system is highly interactive and dynamic.

## Author Contributions

MF, HB, and VM are responsible for the conception, design of the study, as well as for interpretation of the data, drafting the work and revising it critically for intellectual content, and final approval of the version to be published. MF and VM are responsible for data acquisition and MF is responsible for data analysis. MF, HB, and VM are in agreement to be accountable for all aspects of the work in ensuring that questions related to the accuracy or integrity of any part of the work are appropriately investigated and resolved.

## Conflict of Interest Statement

The authors declare that the research was conducted in the absence of any commercial or financial relationships that could be construed as a potential conflict of interest.
