# Supplementary material for: Phonotactic Constraints Are Activated across Languages in Bilinguals
Source: Front Psychol. 2016 May 18;7:702. doi: 10.3389/fpsyg.2016.00702 (PMC4870387; doi:10.3389/fpsyg.2016.00702)
Supplement: Supplementary file 1 [file Data_Sheet_1.DOCX]

Appendix A. Lexical characteristics of prime stimuli, mean (SD). (All *p*s > .05.)

| *Characteristic* | *Cognate prime* | *Non-cognate prime* | *Control* |
| --- | --- | --- | --- |
| English Letters | 6.10 (1.12) | 6.00 (1.53) | 6.47 (1.17) |
| Spanish Letters | 7.50 (1.22) | 7.20 (2.40) | 7.37 (2.71) |
| English Syllables | 1.67 (0.55) | 1.40 (0.50) | 1.63 (0.61) |
| Spanish Syllables | 3.20 (0.55) | 3.03 (1.00) | 3.00 (1.08) |
| ^🞆^English Frequency | 20.89 (31.75) | 14.73 (18.91) | 29.73 (56.97) |
| ^🞆^Spanish Frequency | 23.09 (36.21) | 17.14 (38.24) | 41.33 (82.13) |
| ^🞆^Orthographic Neighbors | 2.53 (3.32) | 3.00 (2.99) | 3.57 (3.35) |
| ^🞆^Phonological Neighbors | 4.03 (5.23) | 5.67 (4.73) | 6.23 (5.72) |
| ^•^Number of Phonemes | 5.57 (1.17) | 4.93 (1.31) | 5.03 (0.93) |
| ^🞆^CLEARPOND (Marian, Bartolotti, Chabal, and Shook, 2012): SUBTLEX frequency in English and in Spanish, orthographic neighborhood size, phonological neighborhood size.  ^•^English Lexicon Project (Balota et al., 2007): number of phonemes. | | | |

Appendix B: Lexical characteristics of target stimuli, mean (SD).

| *Characteristic* | *Phono constraint + form Non-word* | *Phono constraint only*  *Non-word* | *Non-word control* | *Word control* |
| --- | --- | --- | --- | --- |
| English Letters | 7.97 (1.25) | 7.97 (1.25) | 7.97 (1.25) | 7.97 (1.25) |
| ^•^Bigram Sum | 18295.87 (5383.68) | 16593.90 (3800.36) | 1648.00 (4047.21) | 16414.77 (3784.89) |
| ^•^Eng. Bigram Mean | 2617.00 (505.73) | 2449.34 (684.87) | 2419.71 (657.53) | 2370.00 (449.41) |
| ^•^Bigram Position | 1591.07 (673.44) | 1748.63 (537.86) | 1875.47 (490.77) | 1954.70 (813.22) |
| ^•^LDT RT* | 876.37 (75.95) | 862.71 (100.30) | 852.54 (109.83) | 737.52 (70.39) |
| ^•^LDT Zscore* | -0.32 (0.25) | -0.29 (0.31) | -0.24 (0.30) | -0.18 (0.21) |
| ^•^LDT SD | 324.09 (77.26) | 306.82 (73.88) | 297.60 (87.02) | 254.55 (65.42) |
| ^•^Observances | 29.68 (3.18) | 31.38 (3.07) | 29.86 (3.05) | 30.60 (3.11) |
| ^•^LDT ACC | 0.88 (0.09) | 0.91 (0.09) | 0.89 (0.12) | 0.93 (0.09) |
| ^🞆^Ortho. Neighbors | 1.07 (0.25) | 1.10 (0.40) | 1.20 (1.10) | 1.00 (0.00) |
| ^🞆^Total Neighbors | 0.73 (0.45) | 0.79 (0.90) | 0.97 (1.10) | 0.73 (0.64) |
| **p* < .05.  ^•^English Lexicon Project (Balota et al., 2007): summed bigram frequency, average bigram frequency, summed bigram frequency by position, mean lexical decision time latency raw (LDT RT), mean lexical decision time standardized latency (LDT Zscore), standard deviation of lexical decision time latencies (LDT SD), number of observances, mean lexical decision accuracy (LDT ACC)  ^🞆^CLEARPOND (Marian, Bartolotti, Chabal, and Shook, 2012): orthographic neighborhood size, total neighborhood density | | | | |
